# *Nornalup*, a new genus of pselaphine beetle from southwestern Australia (Coleoptera, Staphylinidae, Pselaphinae, Faronitae)

**DOI:** 10.3897/zookeys.695.19906

**Published:** 2017-09-05

**Authors:** Jong-Seok Park, Donald S. Chandler

**Affiliations:** 1 S1-5 302, Major in Biology, Chungbuk National University, 1 Chungdae-ro, Seowon-gu, Cheongju-si, Chungbuk-do 28644, South Korea; 2 Department of Biological Sciences, University of New Hampshire, Durham, NH 03824

**Keywords:** Biodiversity, biogeography, Faronini, taxonomy, Western Australia

## Abstract

A new genus and three new species of the southwestern Australian pselaphine beetles belonging to the supertribe Faronitae are described: *Nornalup* Park & Chandler, **gen. n.**, based on *Nornalup afoveatus* Park & Chandler, **sp. n.**, *Nornalup quadratus* Park & Chandler, **sp. n.**, and *Nornalup minusculus* Park & Chandler, **sp. n.** Illustrations of their habitus and major diagnostic characters are provided, as well as distribution maps and a key to species.

## Introduction

Two faronite genera, *Sagola* Sharp, 1874 with nine species and *Logasa* Chandler, 2001 with three species are known from Australia (Chandler 2001). The former genus includes 131 New Zealand species, and is considered to be a paraphyletic assemblage of species (Chandler 2001). A revision of the New Zealand fauna has been completed by Park and Carlton (2014a–b, 2015a–e).

In the initial steps of revising the Australian faronite fauna, which includes numerous undescribed species (Chandler 2001), it was found that three undescribed species form a morphologically distinctive group. These species are characterized by extremely large eyes, a deep and anteriorly open frontal sulcus, abdominal tergite IV is 1.5 times longer than V, and they also have a different thoracic foveal system from those of the other Australian groups. Foveal patterns have been used extensively for characterizing genera of Pselaphinae (Grigarick and Schuster 1980; Chandler 2001; Park and Carlton 2014a–b, 2015a–e).

## Materials and methods

Thirty-six specimens were examined from the Field Museum of Natural History (FMNH), Chicago, Illinois, USA, and the Donald S. Chandler Collection (DSC), Durham, New Hampshire, USA. Six specimens were mounted on permanent slides to aid in observation of the internal characters and the fine external characters that are not apparent when using a dissecting microscope. Permanent microscopic slides were prepared using the techniques described by Hanley and Ashe (2003). Terminology for the foveal system follows Chandler (2001). Geographical coordinates are reported in Degrees and Decimal Minutes (**DDM**) format. Holotypes are deposited in the Western Australian Museum (**WAM**), Perth, Western Australia, Australia, and paratypes are deposited in the Field Museum of Natural History, the Western Australian Museum, the Australian National Insect Collection (**ANIC**), Canberra, ACT, Australia, and the Chungbuk National University Insect Collection (**CBNUIC**), Cheongju-si, Chungbuk-do, South Korea (indicated parenthetically). Specimen label data for the holotypes is transcribed verbatim. Data for paratypes are standardized for consistency. The map of Australia is created from SimpleMappr (Shorthouse 2010) and was subsequently modified.

## Systematics

### Nornalup

Taxon classificationAnimaliaColeopteraStaphylinidae

Park & Chandler gen. n.

http://zoobank.org/691083BF-7137-48CF-BB87-7F735392CFCD

#### Type species.

*Nornalup afoveatus* Park and Chandler, sp. n., herein designated.

#### Diagnosis.

**Figure 1.**
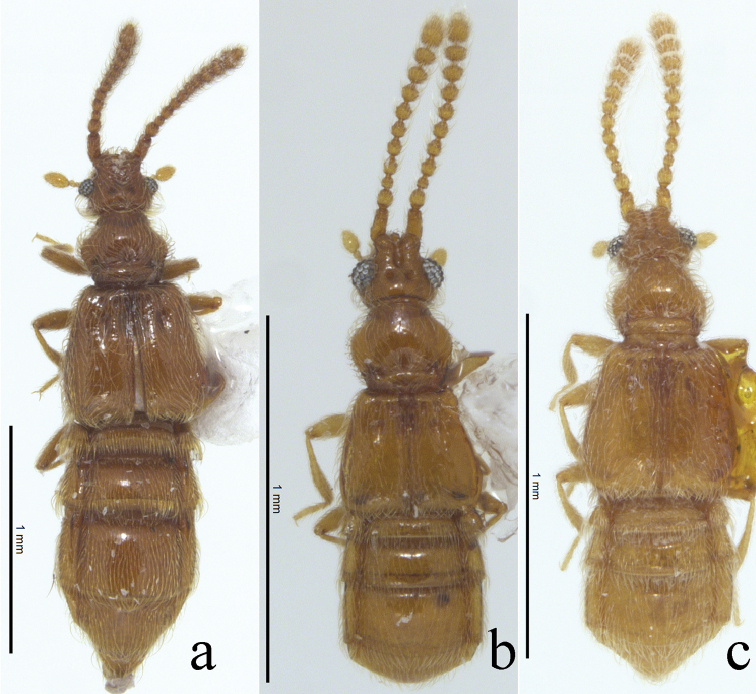
Habiti, dorsal view. **a** *Nornalup afoveatus* sp. n. **b** *N. quadratus* sp. n. **c** *N. minusculus* sp. n. Scale bars: 1 mm.

**Figure 2.**
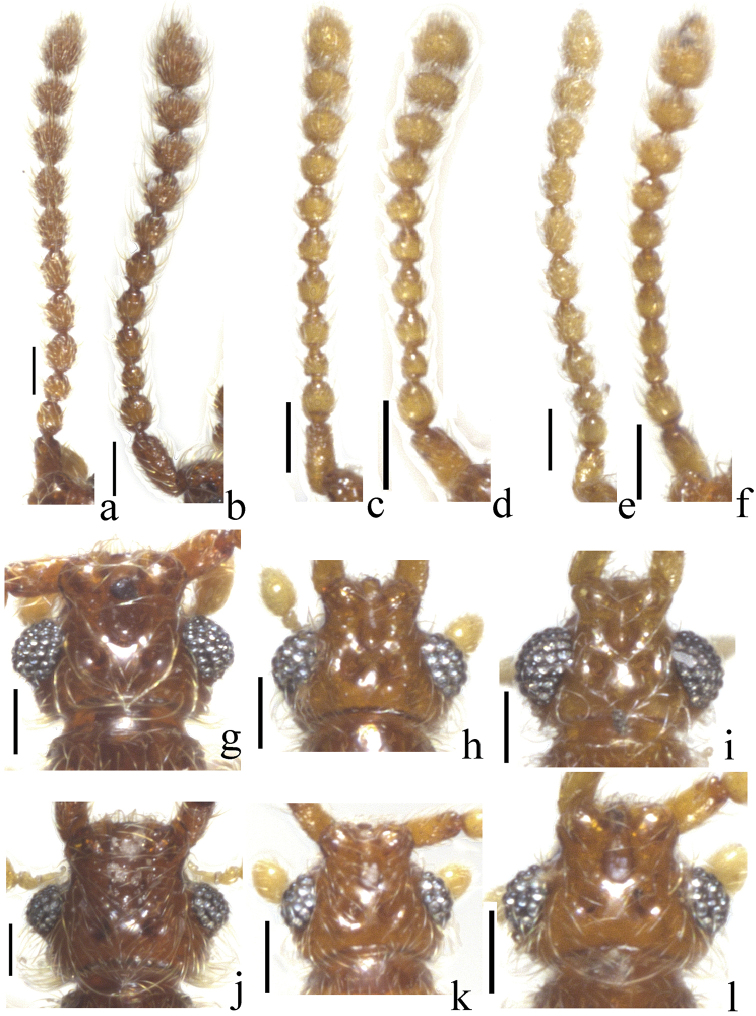
Antennae of *Nornalup afoveatus* sp. n. **a** male **b** female. Antennae of *N. quadratus* sp. n. **c** male **d** female. Antennae of *N. minusculus* sp. n. **e** male **f** female. Male heads, dorsal view **g** *N. afoveatus* sp. n. **h** *N. quadratus* sp. n. **i** *N. minusculus* sp. n. Female heads, dorsal view **j** *N. afoveatus* sp. n. **k** *N. quadratus* sp. n. **l** *N. minusculus* sp. n. Scale bars: 0.1 mm.

**Figure 3.**
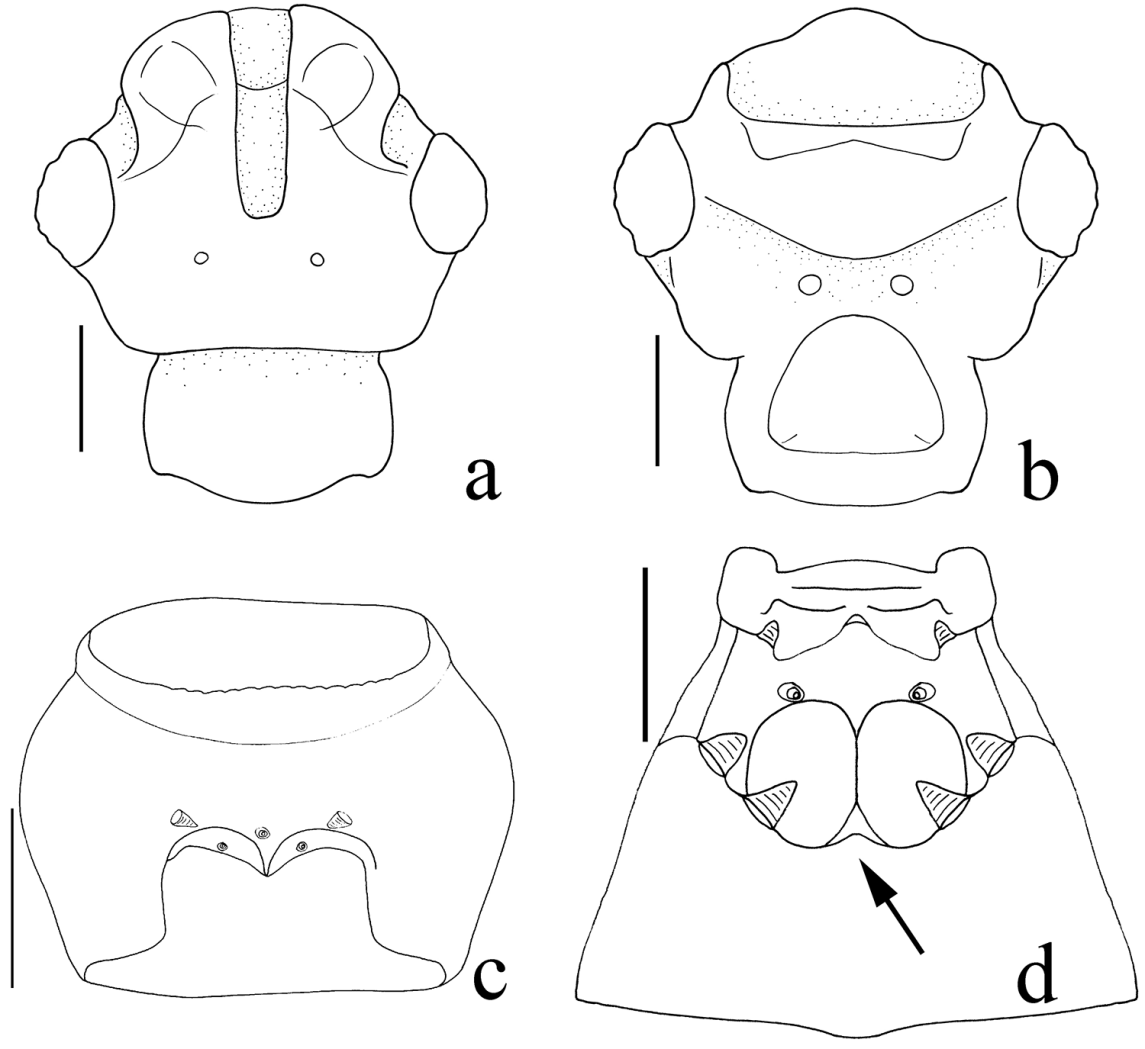
Heads of *Nornalup afoveatus* sp. n. **a** dorsal view **b** ventral view. *N. quadratus* sp. n. **c** prosternum, ventral view **d** meso- and metaventrite, ventral view. Scale bars: 0.1 mm.

**Figure 4.**
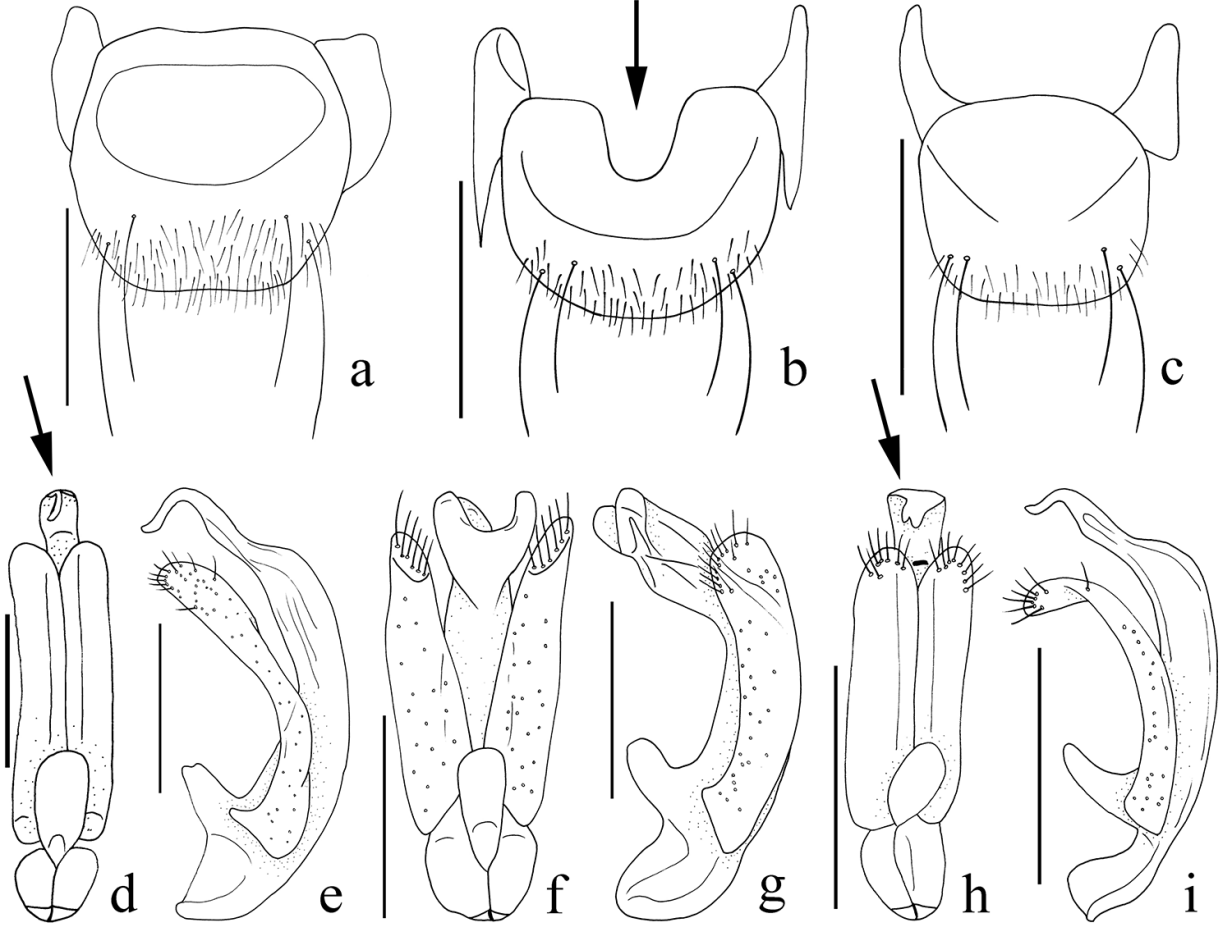
Female abdominal sternites IX, ventral view. **a** *Nornalup afoveatus* sp. n. **b** *N. quadratus* sp. n. **c** *N. minusculus* sp. n. Aedeagi of *N. afoveatus* sp. n. **d** dorsal view **e** lateral view. Aedeagi of *N. quadratus* sp. n. **f** dorsal view **g** lateral view. Aedeagi of *N. minusculus* sp. n. **h** dorsal view **i** lateral view. Scale bars: 0.1 mm.

**Figure 5.**
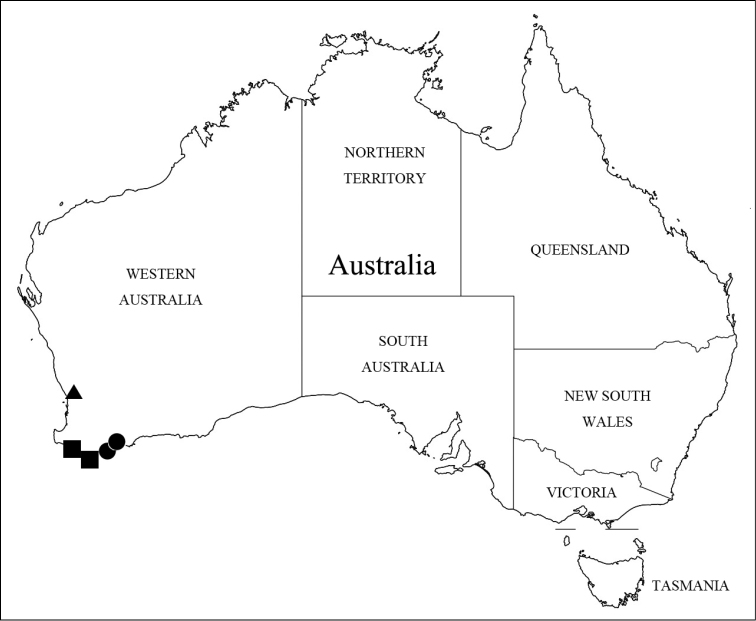
Known collection localities of *Nornalup* gen. n. *N. afoveatus* sp. n.: squares; *N. quadratus* sp. n.: triangle; *N. minusculus* sp. n.: circles.

Members of this genus are easily separated from other faronite genera by the following combination of characters: rostrum separated by distinct frontal sulcus (Fig. 3a); ventral surface of head swollen (Fig. 3b); eyes extremely large, longer than length of temples (Fig. 2g–l); frontal sulcus deep and wide, open anteriorly (Fig. 3a); mesoventrite with lateral mesosternal fovea and promesocoxal fovea (Fig. 3d); metaventrite with or without median metasternal fovea (Fig. 3d: arrow); abdominal length of tergite and sternite VI approximately 1.5 times longer than V (Fig. 1); female sternite IX bearing two pairs of long setae (Fig. 4a–c); species only known from Western Australia (Fig. 5).

#### Description.

Small body size, 1.1–2.5 mm (Fig. 1). Body yellowish to reddish-brown (Fig. 1). Head. Triangular with extremely large eyes, widest across eyes (Fig. 2g–l). Gular area convex (Fig. 3b). Male antennomeres longer than those of female. Male and female antennomeres with tubercles on 4–11 and 8–11, respectively (Fig. 2a–f). Frontal sulcus deep and wide, open anteriorly (Fig. 3a). *Thorax*. Prosternum as long as wide, widest at midpoint of prosternum (Fig. 3c). Prosternum with lateral procoxal and median procoxal fovea (Fig. 3c). Meso- and metathorax trapezoidal, longer than wide (Fig. 3d). Mesoventrite with lateral mesosternal, promesocoxal and lateral mesocoxal foveae (Fig. 3d). Metaventrite with lateral metasternal foveae (Fig. 3d). *Abdomen*. Length of tergite and sternite VI approximately 1.5 times longer than V (Fig. 1). *Aedeagus*. Median lobe longer than parameres (Fig. 4d–i). Phallobase rounded (Fig. 4d–i). Parameres symmetrical, as wide as median lobe, bearing setae at apex (Fig. 4d–i).

#### Etymology.

*Nornalup* gen. n. is named for Nornalup, one of the national parks where one of the species was collected.

#### Distribution.

Australia.

#### Comments about secondary sexual characters.

Male specimens possess tubercles on antennomeres 4–11, but females have the tubercles on antennomeres 8–11 (Fig. 2a–f). Males have slightly larger eyes (Fig. 2g–l). Male abdominal sternite IX is usually fragile and is partially concealed by sternite VIII, rendering it simple and reduced in appearance. Females possess a more robust, rectangular abdominal sternite IX bearing two pairs of long setae (Fig. 4a–c) that are usually visible in ventral view.

#### Comments about biotic region.

*Nornalup* gen. n. is found at the very southwestern corner of Australia, which is known as a global biodiversity hotspot (Hopper and Gioia 2004). This region has a higher average annual rainfall (300–1200 mm) than the surrounding more internal deserts of the mainland, and is mostly covered by *Eucalytus* forests (Hopper and Gioia 2004). Approximately 740 native vascular plants are known from this area, half of which are endemic (Hopper and Gioia 2004). All species are found in the karri (*E. diversicolor* F.Muell.), tingle (*E. jacksonii* Maiden), and jarrah (*E. marginata* Donn ex Sm.) forests unique to this area, where the distributions of three species do not overlap (Fig. 5).

#### Comments about related taxa.

Based on thoracic foveal system, *Nornalup* gen. n. is closest to the genus *Sagola* Sharp. However, the frontal sulcus (Fig. 3a), abdominal length of tergite and sternite VI (Fig. 1), and form of the male aedeagus are not shared with any species of *Sagola* or other faronite genera. To understand the specific relationship with other faronites, phylogenetic analysis based on morphology and molecular data is needed.

#### Key to species of the genus *Nornalup* gen. n.

**Table:** 

1	Elytra quadrate and flattened (Fig. 1b); metaventrite without median metasternal fovea (Fig. 3d: arrow); female abdominal sternite IX emarginate anteriorly (Fig. 4b: arrow)	***Nornalup quadratus* sp. n.**
–	Elytra longer than wide and convex (Fig. 1a, c); metaventrite with median metasternal fovea; female abdominal sternite IX straight anteriorly (Fig. 4a, c)	**2**
2	(1) Body length longer than 2.0 mm (Fig. 1a); apex of aedeagus with one small lobe (Fig. 4d: arrow); female abdominal sternite IX longer than wide, with oval sculpture (Fig. 4a)	***N. afoveatus* sp. n.**
–	Body length smaller than 2.0 mm (Fig. 1c); apex of aedeagus with two small lobes (Fig. 4h: arrow); female abdominal sternite IX as long as wide, without oval sculpture (Fig. 4c)	***N. minusculus* sp. n.**

### Nornalup afoveatus

Taxon classificationAnimaliaColeopteraStaphylinidae

Park & Chandler sp. n.

http://zoobank.org/A773DD78-B39E-4792-B93E-3A94211F4C8E

Figs 1a , 2a–b , 2g , 2j , 3a–b , 4a , 4d–e , 5

#### Type material.

**Holotype. Australia: Western Australia (WA)**: 1♂, aedeagus dissected and mounted in euparal on clear plastic card, “**Australia: Western Australia**: Walpole-Nornalup N.P., Anderson Rd., near Valley of the Giants Rd., 120m, 34°59.48'S, 116°52.35'E, 2 VIII 2004, tingle-*Allocasuarina*-karri (*Eucalyptus diversicolor*) forest; FMHD#2004-137, berl., leaf & log litter, A. Newton, M. Thayer, et al. 1111”. **Paratypes (n = 14; 7 males, 7 females). Australia: Western Australia**: 1♀ (CBNUIC, slide mounted), Warren N. P., Bicentennial Tree vic., 120 m, 34°29.73'S, 115°58.62'E, 30 VII-10 VIII 2004, kauri forest (*Eucalyptus diversicolor*), flight intercept trap, A. Newton & A. Solodovnikov, FMHD#2004-114, 1105; 1♂ (WAM), same as holotype; 1♂ (ANIC), 2-8 VIII 2004, flight intercept trap, A. Solodovnikov, A. Newton & M. Thayer, FMHD#2004-135, 1111; 1♂ (ANIC), Beedelup N. P., Beedelup Falls Rd., 150 m, 34°25.81'S, 115°53.098'E, 31 VII 2004, jarrah (*Eucalyptus marginata*) forest with *Banksia grandis*, *Xanthorrhoea*, A. Newton, M. Thayer, et al., FMHD#2004-128, 1109; 1♂ (FMNH), Warren N. P., Maidenbush Tr., 60 m, 34°30.515'S, 115°57.411'E, 29 VII 2004, old-growth karri forest (*Eucalyptus diversicolor*), A. Newton & D. Clarke, FMHD#2004-113, 1104; 1♂ (FMNH), Pemberton, The Cascades, 7 VII 1980, fungus & jarrah litter, S. Peck & J. Peck; 1♂ (CBNUIC), Brockman N. P., 23.5 km S Pemberton, 6 XII 1976, bark litter, J. B. Kethley, FMHD#76-5031; 1♂ (WAM), Walpole N. P., 5 km NE Coalbine, 5 XII 1976, fungal mat, J. B. Kethley, FMHD#76-470; 1♀ (FMNH), Beedelup N. P., Walk-though Tree vic., 100 m, 34°25.7'S, 115°58.63'E, 30 VII-10 VIII 2004, karri forest (*Eucalyptus diversicolor*), flight intercept trap, A. Newton & M. Thayer, FMHD#2004-116, 1106; 1♀ (CBNUIC), Brockman N. P., 8 XII 1976, leaf litter, debris u. canopy of karri, J. B. Kethley, FMHD#76-482; 1♀ (FMHD), Walpole N. P., 6 km NE Coalbine, 13 XII 1976, litter base of Red Tingle, J. B. Kethley, FMHD#76-493; 2♀♀ (WAM), Nornalup, Valley of Giants, 21 VI 1980, berl., tingle tree bark, S. Peck & J. Peck; 1♀ (ANIC), Walpole N. P., Collier Rd., 19 VI 1980, berl., tingle tree litter, S. Peck & J. Peck.

#### Diagnosis.

This species can be distinguished from *N. quadratus* sp. n. by the longer elytra (Fig. 1a), larger body length (> 2.0 mm), presence of a median metasternal fovea, and the straight anterior margin of female abdominal sternite IX (Fig. 4a). This species is also separated from *N. minusculus* sp. n. by the greater body length (> 2.0 mm, Fig. 1a).

#### Description.

Length 2.0–2.5 mm (Fig. 1a). *Head.* Male antennomeres 1–2 longer than wide, 3 subquadrate, 4–9 longer than wide, 10 subquadrate (Fig. 2a). Female antennomeres 1–2 longer than wide, 3 subquadrate, 4–6 longer than wide, 7–9 subquadrate, 10 weakly transverse (Fig. 2b). *Thorax.* Elytra rectangular and longer than wide (Fig. 1a). Hind wings fully developed. Metaventrite with median metasternal fovea. *Abdomen*. Female abdominal sternite IX with straight anterior margin (Fig. 4a). *Aedeagus.* Apex of male aedeagus with one small lobe, bended as L-shape in lateral view (Fig. 4e).

#### Distribution.

Western Australia (Fig. 5: squares).

#### Habitat.

Specimens of this species were collected using flight intercept traps, or by sifting leaf, bark, or fungus litter in *Eucalyptus* forests.

### Nornalup quadratus

Taxon classificationAnimaliaColeopteraStaphylinidae

Park & Chandler sp. n.

http://zoobank.org/7A39C58D-F5CE-4A23-885C-870F86CE4BC3

(Figs 1b , 2c–d , 2h , 2k , 3c–d , 4b , 4f–g , 5)

#### Type material.

**Holotype. Australia: Western Australia (WA)**: 1♂, aedeagus dissected and mounted in euparal on clear plastic card, “**Australia: Western Australia**: Avon Valley N.P., 1.3 km from entrance, 420m, 31°38.79'S, 116°17.94'E, 27 VII 2004, marris-jarrah (*Eucalyptus calophylla*-*E. marginata*) woodland; FMHD#2004-106, berl., leaf & log litter, A. Newton, D. Clarke, A. Solodovnikov 1102”. **Paratypes (n = 8; 4 males, 4 females). Australia: Western Australia**: 2♂♂ 2♀♀ (1♂ 1♀FMNH, 1♂ 1♀CBNUIC, 1♀ slide mounted), Avon Valley N. P., 1.3 km from entrance, 420 m, 31°38.79'S, 116°17.94'E, 27 VII-13 VIII 2004, marris-jarrah (*Eucalyptus calophylla*-*E. marginata*) woodland, flight intercept trap, A. Newton & M. Thayer, FMHD#2004-103, 1102; 1♀ (FMNH, slide mounted), 27 VII 2004, berl., *Banksia grandis* litter, M. Thayer, FMHD#2004-105, 1102; 2♂♂ 1♀ (1♂ 1♀WAM, 1♀ANIC, 1♂ slide mounted), same as holotype.

#### Diagnosis.

This species can be distinguished from *N. afoveatus* sp. n. by the quadrate elytra (Fig. 1b), shorter body length (< 2.0 mm, Fig. 1b), lack of a median metasternal fovea (Fig. 3d: arrow), and emarginate anterior margin of female abdominal sternite IX (Fig. 4b: arrow). This species is also separated from *N. minusculus* sp. n. by the quadrate elytra (Fig. 1b), lack of a median metasternal fovea (Fig. 3d: arrow), and the emarginate anterior margin of female abdominal sternite IX (Fig. 4b).

#### Description.

Length 1.1–1.5 mm (Fig. 1b). *Head.* Male antennomeres 1–2 longer than wide, 3 subquadrate, 4–6 longer than wide, 7–8 subquadrate, 9–10 weakly transverse (Fig. 2c). Female antennomeres 1–2 longer than wide, 3 subquadrate, 4–5 longer than wide, 6–8 subquadrate, 9–10 weakly transverse (Fig. 2d). *Thorax.* Elytra subquadrate (Fig. 1b). Hind wings reduced, half size of other species. Metaventrite without median metasternal fovea (Fig. 3d, arrow). *Abdomen*. Female abdominal sternite IX with emarginate anterior margin (Fig. 4b). *Aedeagus*. Apical lobe of median lobe divided into two lobes as U-shape (Fig. 4f).

#### Distribution.

Western Australia (Fig. 5: triangle).

#### Habitat.

Specimens of this species were collected using flight intercept traps, or by sifting leaf, log, or *Banksia grandis* litter in *Eucalyptus* forests.

#### Comments.

Both sexes of this species have the hind wings approximately half normal size when compared to the other species. However, four specimens were collected by flight intercept trap, so we speculate that this species still has the ability to fly.

### Nornalup minusculus

Taxon classificationAnimaliaColeopteraStaphylinidae

Park & Chandler sp. n.

http://zoobank.org/E094EE5F-7D6B-4246-A02C-6170213E37D1

(Figs 1c , 2e–f , 2i , 2l , 4c , 4h–i , 5)

#### Type material.

**Holotype. Australia: Western Australia (WA)**: 1♂, aedeagus dissected and mounted in euparal on clear plastic card, “**Australia: Western Australia**: Porongurup N.P., Nancy Peak Tr., Morgan’s View to The Pass, 450–600m, 34°40.8'S, 117°51.65'E, 6 VIII 2004, *Eucalyptus*; FMHD#2004-149, berl., leaf & log litter, Clarke & Grimbacher 1118”. **Paratypes (n = 9; 3 males, 6 females). Australia: Western Australia**: 1♂ 1♀ (CBNUIC), 40 km ESE Manjimup, 15 VII 1980, jarrah forest litter, S. Peck & J. Peck; 1♂ (WAM), 83 km NE Albany, Stirling Range N. P., Toolbrunup Peak, 700 m, 27 XII 1976, litter at stream edge. u. marri, below 1st talus, J. B. Kethley, FMHD#76-537; 1♂ (FMNH), Giant Tingle Area, 8 km NE Walpole, 19 XII 1976, Karri & Acacia l., J. B. Kethley, FMHD#76-514; 1♀ (FMNH), Porongurup N. P., Wansborough Walk at The Pass, 450 m, 34°40.69'S, 117°51.245'E, 6 VIII 2004, karri forest (*Eucalyptus diversicolor*), mostly young-growth, berl., leaf & log litter, A. Newton & M. Thayer, FMHD#2004-147, 1116; 2♀♀ (WAM, 1♀ slide mounted), Stirling Range N. P., Toolbrunup Peak Tr., 480–520 m, 34°23.4'S, 118°03.3'E, 5 VIII 2004, *Eucalyptus* forest & mallee, berl., lead & log litter, D. Clarke & Grimbacher, FMHD#2004-146, 1115; 2♀♀ (ANIC, 1♀ slide mounted), 430–485 m, 34°23.5'S, 118°03.65'E, 5 VIII 2004, mallee *Eucalyptus*, berl., water-washed soil, 0–18 cm, D. Clarke, FMHD#2004-145, 1114; 1♀ (CBNUIC), 43 km E Albany, Two People’s Bay, Mt. Gardner, 150m, 1 I 1977, litter u. *Hibbertia* sp., J. B. Kethley, FMHD#77-88; 1♀ (ANIC), 220m, 1 I 1977, litter u. Marri, J. B. Kethley, FMHD#77-85.

#### Diagnosis.

This species can be distinguished from *N. quadratus* sp. n. by the longer elytra (Fig. 1c), presence of a median metasternal fovea, and the straight anterior margin of female abdominal sternite IX (Fig. 4c). This species is also separated from *N. afoveatus* sp. n. by its smaller body length (< 2.0 mm, Fig. 1c).

#### Description.

Length 1.2–1.6 mm (Fig. 1c). *Head.* Male antennomeres 1–2 longer than wide, 3 subquadrate, 4–9 longer than wide, 10 subquadrate (Fig. 2e). Female antennomeres 1–2 longer than wide, 3 subquadrate, 4–6 longer than wide, 7–8 subquadrate, 9–10 weakly transverse (Fig. 2f). *Thorax.* Elytra rectangular and longer than wide (Fig. 1c). Hind wings fully developed. Metaventrite with median metasternal fovea. *Abdomen*. Female abdominal sternite IX with straight anterior margin (Fig. 4c). *Aedeagus.* Apex of male aedeagus with two small lobes, bent into an L-shape in lateral view (Fig. 4i).

#### Distribution.

Western Australia (Fig. 5: circles).

#### Habitat.

Most specimens of this species were collected by sifting leaf and log litter, with one taken from water-washed soil in *Eucalyptus* forests.

## Supplementary Material

XML Treatment for Nornalup

XML Treatment for Nornalup afoveatus

XML Treatment for Nornalup quadratus

XML Treatment for Nornalup minusculus
